# Birth size, growth trajectory and later cardio-metabolic risk

**DOI:** 10.3389/fendo.2023.1187261

**Published:** 2023-06-05

**Authors:** Chiara Cauzzo, Valentina Chiavaroli, Susanna Di Valerio, Francesco Chiarelli

**Affiliations:** ^1^ Department of Pediatrics, University of Chieti, Chieti, Italy; ^2^ Neonatal Intensive Care Unit, Pescara Public Hospital, Pescara, Italy

**Keywords:** intrauterine growth, small for gestational age, birth weight, preterm, cardiovascular risk

## Abstract

There is increasing evidence of a strong association between intrauterine growth and subsequent development of chronic disease in adult life. Birth size and growth trajectory have been demonstrated to have an impact on cardio-metabolic health, both in childhood and adult life. Hence, careful observation of the children’s growth pattern, starting from the intrauterine period and the first years of life, should be emphasized to detect the possible onset of cardio-metabolic sequelae. This allows to intervene on them as soon as they are detected, first of all through lifestyle interventions, whose efficacy seems to be higher when they are started early. Recent papers suggest that prematurity may constitute an independent risk factor for the development of cardiovascular disease and metabolic syndrome, regardless of birth weight. The purpose of the present review is to examine and summarize the available knowledge about the dynamic association between intrauterine and postnatal growth and cardio-metabolic risk, from childhood to adulthood.

## 1 Introduction

The switch in the worldwide pattern of diseases from infectious illnesses towards chronic disorders has been of special concern for several decades in developed countries, but it is becoming relevant also in developing nations, which are facing an epidemiological transition towards an emerging epidemic of chronic diseases (1). Of note, it is becoming increasingly evident the strong connection between birth weight and subsequent development of chronic diseases in adult life (2–5). Namely, there is evidence from several studies that individuals born small for gestational age (SGA) are more likely to present cardio-metabolic complications in later life (6–8). Birth weight is not only determined by genetic factors, but it is also influenced by the prenatal environment (9, 10). It has been reported that unfavourable intrauterine environment and compromised fetal growth play a part in the development of atherosclerosis and adult cardiovascular disease (1). Therefore, greater attention should be given to a healthy lifestyle for women, especially in the months before conception and during pregnancy (11, 12). There is recent evidence that also prematurity may be an independent risk factor for the onset of cardiovascular disease and metabolic syndrome, regardless of birth weight (13).

While some studies indicate intrauterine undernutrition as the main factor in the determination of chronic disease, other authors highlight the crucial role of growth trajectory in the postnatal period as the most important determinant in later expression of chronic diseases, primarily cardiovascular and cerebrovascular (14–18). Likewise, careful observation of the children’s growth pattern, since the first years of life, should be emphasized. Furthermore, it is important to monitor the onset of the cardio-metabolic consequences linked to those growth patterns in order to promote strategies that contribute to health improvement (19, 20). The challenge is to intervene on cardio-metabolic risk factors as soon as they are detected, first of all through lifestyle interventions, whose efficacy seems to be higher when they are started early (21, 22).

Therefore, the purpose of the present review is to examine and abridge the available knowledge about the dynamic association between birth size as well as growth trajectory and cardio-metabolic risk in the subsequent extrauterine life, starting from childhood into adulthood.

An ample literature search was conducted through the main databases, including PubMed, UpToDate and Scopus, in order to examine the available data about definitions, pathogenesis, and consequences of being born SGA and/or preterm as well as the clinical management and approach to prevent and/or postpone future health problems. The following search terms were used: preterm birth, premature, SGA, birth size, cardio-metabolic risk, cardiovascular risk, diabetes and hypertension. Reference list of retrieved records was also checked out. The search was limited to the English language papers and was updated on May 1, 2023.

## 2 Definitions

The terms used in this review are the following: intrauterine growth restriction (IUGR), SGA, prematurity, low-, very low- and extremely low-birth weight (LBW), catch-up growth, overweight, obesity, and metabolic syndrome.

Overall, birth weight is influenced by a variety of determinants, including genetics, ethnicity, maternal nutrition before and during pregnancy, maternal obesity, smoking, and diabetes (23). The mechanisms responsible for impaired fetal growth can be classified as fetal, utero-placental, and maternal (such as malnutrition, low maternal body mass index (BMI) before pregnancy and insufficient gestational weight gain) (7, 24).

Although IUGR is often used as a synonym for SGA, this acronym refers to a condition where the fetus is not able to reach its growth potential because of underlying pathological conditions (7). Thus, the term IUGR should be used only in reference to the fetus and can be estimated from fetal sonographic measurements, through which a diminished growth velocity can be detected on serial ultrasounds (25). This growth pattern can be distinguished in early IUGR, when recognized before 32 weeks of gestation, and in late IUGR, if detected at 32 weeks of gestation or beyond (25). Many fetuses with IUGR may be born SGA, depending on timing of the intrauterine insult and its severity, but not all of them. On the other hand, many SGA newborns have not experienced IUGR (24, 26).

The term SGA is used to describe infants having a birth weight and/or length below the expected range for gestational age. While neonatologists tipically define SGA as a newborn with birth weight lower than the 10^th^ percentile for gestational age (24), for pediatric endocrinologists SGA is defined as a birth weight and/or length at least 2 standard deviations (SDs) beneath the mean for gestational age and sex, derived from reference populations data (24, 27). Namely, the definition of the 10^th^ percentile is relevant to evaluate morbidity and mortality in the neonatal period (24, 28), while the cut-off point of -2 SDs may help recognizing those infants who need careful and ongoing growth monitoring (22, 29). Infants can be subcategorized into the following three groups: SGA for weight, SGA for length and SGA for weight and length (26, 30). In order to obtain an accurate classification of SGA, it is recommended to use national growth charts or, as an alternative, the most appropriate ones for the region- and ethnic-specific population (26).

According to the World Health Organization (WHO), preterm birth is defined as a birth happening before 37 completed weeks of gestation, or less than 259 days after the first day of the last menstrual period preceding the pregnancy (31). Preterm birth can be further categorized into extremely preterm (< 28 weeks), very preterm (28 to < 32 weeks), and moderate (32 to < 34 weeks) to late preterm (34 to < 37 weeks) (32). The worldwide rate of preterm birth is about 11%, corresponding approximately to 15 million babies born preterm every year (32, 33). Preterm birth can be intended as an adverse pregnancy outcome (where the fetus is unable to achieve the growth potential inside maternal uterus) or a preferred outcome (where a miscarriage or non-viable prematurity has been avoided). Unfortunately, a proportion of babies can be born preterm even in low-risk pregnancies of healthy women (34).

WHO defines LBW as a weight at birth < 2500 g. This definition embraces both preterm infants who usually have appropriate size for their gestational age and infants born at term with poor birth weight (24). Very low- and extremely low-birth weight are specified as birth weight < 1500 and < 1000 g, respectively (22).

Catch-up growth is defined as an accelerated growth velocity in weight and/or height during early life that compensates the poor intrauterine growth in children born SGA. Indeed, approximately 90% of children born SGA undergo catch-up growth before the age of two (35, 36). This compensatory growth has been linked to positive effects in children born SGA, mainly on cognitive abilities and adult height (35). However, recent literature is increasingly focusing on the idea that growth patterns in the first years of life might influence long-term health (37–39). Catch-up growth, primarily in weight, has been proven to have an effect on cardio-metabolic risk factors, including overweight, obesity, and insulin resistance both in childhood and, to a greater extent, in adulthood, independently of birth weight (20, 35, 36). Rapid catch-up growth in the postnatal period is more common in newborns with LBW, which makes them more prone to chronic diseases in adult life (11). These findings underline the importance of a regular growth monitoring in these children.

WHO’s definition of overweight and obesity in children younger than 5 years refers to a weight-for-height index > 2 and 3 SDs above the WHO Child Growth Standards median, respectively. In older children and adolescents the definition is based on the BMI-for-age score, distinguishing overweight when > 1 and obesity when > 2 SDs over the WHO Growth Reference median. For adults, overweight and obesity are defined by WHO as a BMI ≥ 25 kg/m^2^ and 30 kg/m^2^, respectively (40).

Lastly, according to Cook’s criteria, metabolic syndrome is defined as the co-existence of three or more of the five following components: abdominal circumference ≥ 90^th^ percentile, blood pressure ≥ 90^th^ percentile, fasting glycemia ≥ 100 mg/dl, HDL cholesterol ≤ 40 mg/dl and triglycerides ≥ 110 mg/dl (41). In 2014 a definition of metabolic syndrome for prepubertal children was proposed by the IDEFICS study, which used values obtained from 18.745 European children to determine age-specific and sex-specific percentiles (height-specific when considering blood pressure), from which to establish cutoffs for the metabolic syndrome components in children with age between 2 and 11 years: waist circumference (≥ 90^th^ percentile); triglycerides (≥ 90^th^ percentile); HDL cholesterol (≤ 10^th^ percentile); blood pressure (systolic ≥ 90^th^ percentile or diastolic ≥ 90^th^ percentile); glucose metabolism (insulin ≥ 90^th^ percentile or fasting glucose ≥ 90^th^ percentile). In accordance with this last definition, a careful and strict follow-up is required when three or more of the risk factors overcome the 90^th^ percentile (≤ 10^th^ percentile for HDL cholesterol); intervention is recommended if three or more risk factors exceed the 95^th^ percentile (≤ 5^th^ percentile for HDL cholesterol) (42, 43).

## 3 Pathogenesis of cardio-metabolic sequelae

**Figure 1:**
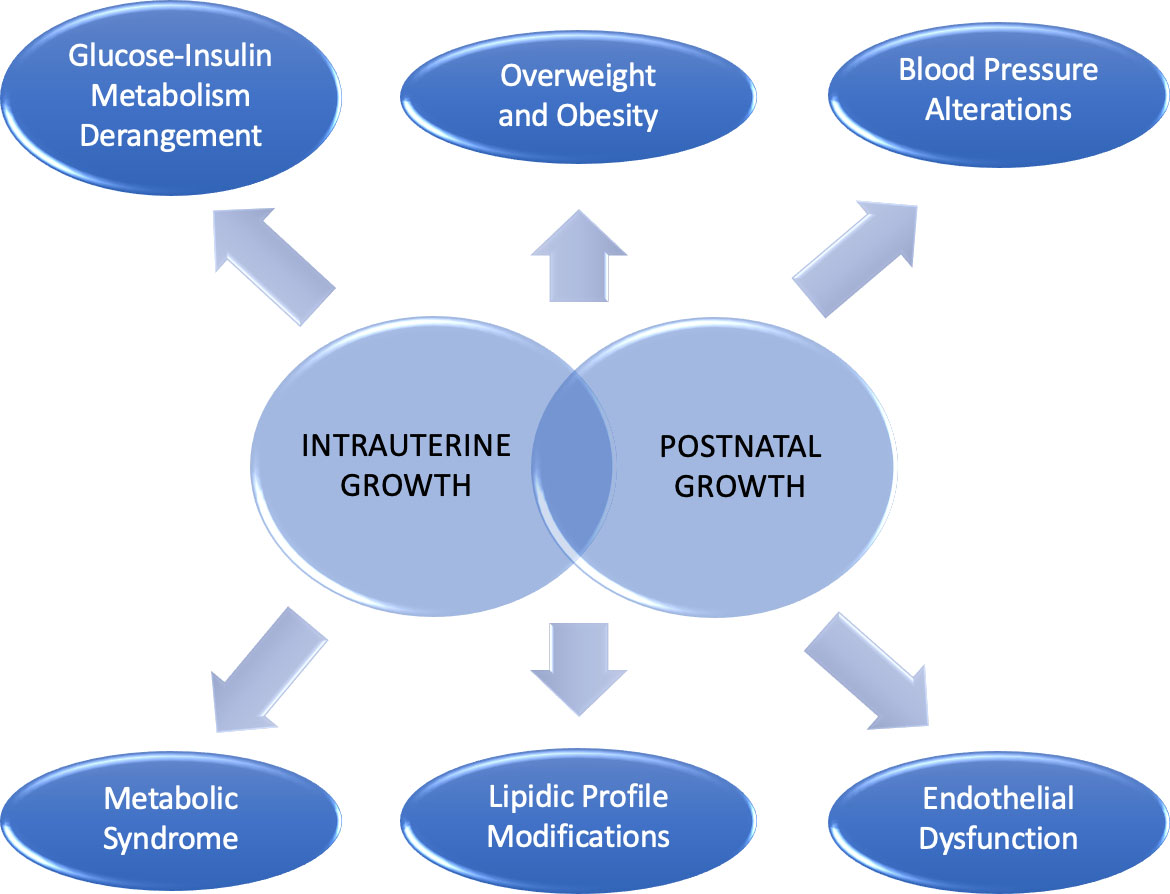
Cardio-metabolic consequences linked to intrauterine and postnatal growth.

There is evidence that birth size and growth trajectory can be related to an increased cardio-metabolic risk. Namely, a link has been shown with the following health issues: glucose-insulin metabolism derangement, overweight/obesity, blood pressure alterations, endothelial dysfunction, lipidic profile modifications, and metabolic syndrome (22) (**Figure 1**).

Barker was the first epidemiologist who suggested the correlation between LBW and cardiovascular disease and metabolic syndrome in later life (22, 44). Several hypotheses have been proposed to clarify this unfavorable association. The “fetal origins hypothesis” was formulated as a theory for the origin of type 2 diabetes (T2D), suggesting that glucose and insulin metabolism are programmed already in fetal life by factors able to affect also fetal growth, primarily maternal nutrition (45). According to the “fetal insulin hypothesis” genetically determined insulin resistance results in an impaired insulin-mediated growth in the fetus and in the development of insulin resistance in adulthood. Therefore, LBW, insulin resistance, glucose intolerance, diabetes and hypertension could all be phenotypes of the same insulin-resistant genotype (46). Indeed, the correlation observed between monogenic diseases that alter glucose tolerance or increase insulin resistance with impaired fetal growth supports the previous theory (46, 47). Afterwards, the “thrifty phenotype hypothesis” proposed that when a fetus endures insufficient nutrition during gestation the growth and development of vital organs, such as the brain, is guaranteed at the expense of other “less noble” tissues, such as the muscle and the endocrine pancreas. Of importance, the metabolism is able to adapt to a condition of limited nutrition, being programmed to take advantage when facing similar conditions in postnatal life (48). This last hypothesis was extended by the “predictive adaptive response hypothesis”: the fetus dynamically interacts with the environment and adapts to it (“developmental plasticity”) in order to survive when exposed to hostile intrauterine conditions (3). However, this “metabolic programming” becomes unfavorable when the fetus is exposed to excessive nutrition postnatally (49). Lastly, in the “rapid catch-up growth hypothesis”, LBW itself is not seen as a risk factor for chronic diseases as only SGA newborns who experience a rapid catch-up growth during the first years of life show an increased cardio-metabolic risk (50–52). Thus, the “metabolic programming” for later diseases starts before birth and continues throughout childhood (53).

Epigenetic changes (e.g., DNA methylation, histone modification, and noncoding RNAs) have also been proven to exert a pivotal role in the development of cardio-metabolic diseases (54). A recent work hints that alterations at birth in DNA methylation of specific imprinted genes (e.g., PLAGL1, MEST, PEG10, and NNAT DMRs) are correlated with the risk of obesity at the age of 1 and 3 years (55), supporting the findings from a previous study in older children (56). In addition, birth weight has itself been linked to epigenetic modifications. Namely, DNA methylation at several CpG sites at birth has been associated with birth weight, and this association has been partly observed also in childhood (57).

Overall, it can be assumed that the susceptibility to cardio-metabolic disease originates from a combination of both genetic and environmental factors.

## 4 Glucose-insulin metabolism derangement

The correlation between birth weight and increased risk of developing T2D has been demonstrated in both children and adults (58, 59) (**Table 1**). Namely, most of the studies reported an inverse relationship of birth weight with: i) fasting glucose and insulin levels; ii) glucose levels two hours after a glucose tolerance test; iii) prevalence of T2D; and iv) insulin resistance, regardless of sex. In addition, these relationships are present whether or not adjustment for current size was carried out, providing indirect support for the “fetal origins hypothesis” (45).

**Table 1:** Glucose-Insulin Metabolism Derangement.

GLUCOSE-INSULIN METABOLISM DERANGEMENT	
•Birth weight is related to increased risk of T2D in children and adults •Children born SGA have lower insulin sensitivity and increased risk of T2D •Accelerated early weight gain increases insulin resistance in childhood and adulthood •Children born preterm have greater insulin resistance and the effect of prematurity is independent of intrauterine growth •Preterm birth is associated with higher HOMA-IR, increased fasting glucose and insulin levels •Adults born prematurely have altered OGTT, increased cardiovascular risk and centralization of fat distribution •Preterm birth may be a risk factor for T1D, while being born SGA seems to be protective for T1D	
*Healthcare professionals should promptly recognize glucose-insulin alterations in subjects born SGA and/or preterm, especially in presence of early weight gain*	

HOMA-IR, HOmeostatic Model Assessment - Insulin Resistance; OGTT, Oral Glucose Tolerance Test; SGA, Small for Gestational Age; T1D, Type-1 Diabetes; T2D, Type-2 Diabetes.

It has been reported that children born SGA have an increased risk of T2D than peers born appropriate-for-gestational-age (AGA) since school-age (59). It is well known that overweight and obesity, mostly in combination with a central distribution of adipose tissue, are associated with hyperinsulinism (60, 61). Nevertheless, glucose metabolism has been found altered independently of fatness in children born SGA (62). Insulin sensitivity, assessed by intravenous glucose tolerance test, has been found lower in SGA children than AGA peers and, as a consequence, the former group compensate by almost tripling the acute insulin response in the attempt to maintain a normal glucose tolerance (62). In children with obesity, being born SGA increases insulin resistance when compared to being born AGA (63). Although several studies have observed an increase in insulin levels and greater insulin resistance in SGA children compared to AGA peers, not all of them have found a consensual increase in fasting glucose levels (64).

Besides birth weight, accelerated early weight gain also contributes to increase the risk of insulin resistance in childhood, with the long-term effects of insulin resistance extending into adulthood (65–69). As a matter of fact, rapid infancy weight gain has been shown to be a strong predictor of fat accumulation during adult life (70), while another study reported only a feeble effect of rapid infancy weight gain on insulin levels in young adulthood (69).

Greater insulin resistance has been found also in children born preterm (71). Namely, 7-year-old children born prematurely have been found to be more insulin-resistant than controls having the same age; the effect of prematurity was independent of intrauterine growth (71). Another study involving 6-year-old children born preterm showed increased basal insulin and C-peptide levels in those with birth weights lower than the 10^th^ percentile (72). In a recent systematic review and meta-analysis, an association between preterm birth and higher homeostatic model assessment for insulin resistance (HOMA-IR) levels, a surrogate marker of insulin resistance, has been observed, together with increased fasting glucose and insulin levels. It was concluded that preterm birth is strongly related with many metabolic syndrome components and cardiovascular disease in adult life, although the results were not differentiated between individuals born AGA and those born SGA (73). It must be emphasized that infants born very preterm differ from full term ones in the postnatal growth curve, which shows an initial slowing of growth speed followed by a later catch-up growth (i.e. at approximately 4 years of age) (74, 75). Higher fasting concentrations of 32-33 split proinsulin, a marker of insulin resistance, have been reported in children born preterm aged 13–16 years with rapid postnatal weight gain compared to those without (76). In adults born prematurely, altered glucose tolerance test and increased risk for cardiovascular disease were reported when compared to adults born at term; however, it must be highlighted that the preterm group included subjects who were born SGA or with LBW (13). Another study, conducted in a quite wide population of individuals born very preterm, demonstrated that accelerated weight gain in the first three months of age predicted higher insulin levels at age 19, although the association was weak. Furthermore, fat accumulation strongly correlated with higher insulin and C-peptide levels as well as increased HOMA-IR at 19 years of age. The impact of adult fat accumulation on these parameters of insulin resistance was found to be conditioned by birth weight SDs (69). In accordance with the previous findings, it has been showed that HOMA-IR (77) was relatively high in men and women born very preterm and that these subjects, already at the age of 19 years, showed some centralization of fat distribution compared with a reference population (69).

Some wide population studies showed that both SGA and preterm birth can be risk factors for T2D onset in children (78, 79). The query of whether adults born very preterm, especially if SGA at birth and subsequently becoming overweight, experience an early onset of T2D is still open and needs to be further evaluated (69, 80).

Of note, while preterm birth has been described as a risk factor for type 1 diabetes (79), being born SGA seems to have a protective effect on this disease onset (81). These observations suggest different repercussions of SGA and preterm birth on glucose-insulin metabolism (82).

Overall, as there is a tracking of glucose-insulin metabolism derangement from childhood to adulthood, healthcare professionals should promptly recognize glucose-insulin alterations in subjects born SGA and/or preterm, especially in presence of early weight gain.

## 5 Overweight and obesity

Overweight and obesity are clearly related to cardio-metabolic risk factors. Thus, early preventive strategies need to be applied given that worldwide the prevalence of overweight and obesity has remarkably increased in the pediatric population, even in early childhood, and many of these preschoolers maintain obesity also in adolescence and adulthood (19, 20) (**Table 2**).

**Table 2:** Overweight and obesity.

OVERWEIGHT AND OBESITY
•Children born SGA have increased risk for obesity, already in childhood and adolescence •Rapid early weight gain predisposes to visceral adiposity •Children born SGA show a reduced total fat mass with normal quantity of visceral fat but lower amount of subcutaneous fat •Visceral fat is associated with IR and metabolic derangement both in children and adolescents •Subcutaneous adipose tissue may have a neutral or even protective effect towards IR •Puberty is a time of hormonal modifications, which may magnify the metabolic abnormalities •In obese adolescents, puberty is an important risk factors for transition from metabolically healthy to unhealthy obesity •A more rapid weight gain after preterm birth is associated with altered body composition in childhood (higher fat mass percentage, fat mass index and waist circumference) •Adults born preterm have a higher percentage of total body fat mass and greater risk for cardiovascular disease
*Overweight and obesity represent a risk for later cardio-metabolic disease* *Regular growth evaluation is mandatory to guarantee early and appropriate interventions* *Counseling in nutrition, physical activity and sleep routine for the whole family*

IR, Insulin Resistance; SGA, Small for Gestational Age.

In 2018 a longitudinal study starting at birth and extended to the whole first decade of life analyzed the connection of birth weight and current size with cardio-metabolic risk factors. The main finding was that the impact of current body size on cardio-metabolic parameters amplified over the years, partially numbing the potential effect of birth weight. Indeed, obesity at 5 years of age was directly related to birth weight, increased by maternal obesity and partially mitigated by breastfeeding, whereas at 10 years of age maternal obesity was the only factor related to obesity in children (20).

Children born SGA have an increased risk for obesity already during childhood and adolescence (7, 83). As there is clear evidence that rapid early weight gain predisposes to visceral adiposity (22), it is mandatory to promptly recognize those children born SGA who experience rapid catch-up growth, as they can develop visceral adiposity since pre-pubertal stage, even without overweight (84).

Furthermore, a reduced total fat mass with a lower amount of subcutaneous fat has been observed in SGA children, determining an increased visceral to subcutaneous fat ratio (85, 86). The role of body composition in cardio-metabolic risk is a relevant point to be addressed. Indeed, considering that regional body fat distribution is a well-known determinant of cardiovascular risk, body composition has acquired a prognostic significance (87). Visceral adipose tissue has been associated with insulin resistance and metabolic syndrome in adults (88) and, compared to subcutaneous fat, visceral fat is related to a less favorable adipokyne and inflammatory profile, leading to a significant reduction in insulin sensitivity (88, 89). Several studies have associated visceral fat to insulin resistance and metabolic derangement both in children and adolescents (90–95). Studies examining subcutaneous adipose tissue independently of total and visceral adipose tissue in obese adolescents showed that its effect may be neutral or even protective towards insulin resistance (90, 95). Indeed, subcutaneous fat may behave as a buffer and prevent lipotoxicity in other tissues, in consonance with the “adipose tissue expandability” hypothesis (88, 89). All these observations underline the importance of considering the impact of body composition in the SGA group.

Of importance, overweight and obesity may develop later in those born SGA (96). Puberty is another time of substantial metabolic and hormonal modifications, which may magnify the metabolic abnormalities. This has remarkable implications for adolescents with obesity, as evidence shows puberty as one of the prominent risk factors for transition from metabolically healthy to unhealthy obesity (97, 98). Notably, many studies reported a strong association between LBW and overweight in adolescence (99), and the risk for obesity remains high even in adult life in individuals born SGA (7, 83).

A more rapid weight gain after preterm birth has been associated with altered body composition (higher fat mass percentage, fat mass index and waist circumference) in childhood (100).

In adults born preterm a higher percentage of total body fat mass and a greater risk for cardiovascular disease have been reported when compared to individuals born at term, although results were not differentiated between adults born SGA and those born AGA (13, 73).

Given that overweight and obesity represent a risk for later cardio-metabolic disease (101), it is essential to carry a regular growth evaluation in these high-risk groups of children to guarantee early and appropriate interventions. As a matter of fact, counseling in nutrition, physical activity and sleep routine addressed to the whole family may improve their future cardio-metabolic health.

## 6 Blood pressure alterations

Unsatisfactory intrauterine growth has been correlated with elevated systolic blood pressure in children, adolescents, and adults (8) (**Table 3**). The relationship between birth weight and blood pressure in childhood may be partly explained by the effect of birth weight on childhood overweight (102), although many authors found an independent correlation between weight at birth and blood pressure during childhood and adolescence (103). Maternal preeclampsia has also been related to higher systolic and diastolic blood pressure in children, and being born SGA after a pre-eclamptic pregnancy increases the risk of developing hypertension in childhood and young adulthood (104, 105). Nevertheless, although at birth blood pressure is related to birth weight, overtime it becomes progressively more dependent on body size, while the impact of birth weight gradually reduces (106, 107). Indeed, current weight has been recognized as the main determinant of childhood hypertension. An early catch-up growth also amplifies the risk of hypertension with onset in childhood (8, 108). As elevated blood pressure in childhood rises the risk for cardiovascular disease in adulthood, it is crucial to provide proper interventions as soon as possible (109, 110).

**Table 3:** Blood Pressure Alterations.

BLOOD PRESSURE ALTERATIONS
•Unsatisfactory intrauterine growth is correlated with elevated SPB in children, adolescents and adults •Maternal preeclampsia is related to higher SBP and DBP in children •Being born SGA after a pre-eclamptic pregnancy increases the risk of hypertension in childhood and young adulthood •At birth BP is related to birth weight, but overtime it becomes progressively more dependent on body size, while the impact of birth weight gradually reduces •Current weight is the main determinant of childhood hypertension •Early catch-up growth also amplifies the risk of hypertension in childhood •Children born very preterm have higher systolic and diastolic mean values and the BP increase is even more meaningful in individuals born preterm who experienced early rapid weight gain •Children born preterm, especially with accelerated weight gain, have higher BP in adolescence •Adults born preterm have elevated BP and increased risk for cardiovascular disease
*Elevated BP in childhood rises the risk for cardiovascular disease in adulthood* *It is crucial to provide proper interventions as soon as possible*

BP, Blood Pressure; DBP, Diastolic Blood Pressure; SBP, Systolic Blood Pressure; SGA, Small for gestational age.

Considering a more long-term risk, a population-based study including over 11.000 women who were born SGA found an increased risk of severe preeclampsia during future pregnancies (26, 111).

Of note, a recent study including toddlers born very preterm demonstrated a significant blood pressure increase in both systolic and diastolic mean values compared to a term-born cohort (112). The worrying detection of these alterations already during the toddler period could indicate an increased risk of cardiovascular diseases in adulthood for this population. Moreover, the blood pressure increase was even more meaningful in individuals born preterm who experienced rapid weight gain in early life (112). Indeed, children born preterm, especially those who experience a more rapid weight gain in childhood, have been found to have higher blood pressure in adolescence (100). In adults born preterm elevated arterial blood pressure and increased risk for cardiovascular disease have been observed when compared to individuals born at term (13).

## 7 Endothelial disfunction and lipidic profile modifications

Some authors have described an association between birth weight and impaired endothelial function in childhood (113, 114) and adulthood (115) (**Table 4**).

**Table 4:** Endothelial disfunction and lipidic profile modifications.

ENDOTHELIAL DISFUNCTION AND LIPIDIC PROFILE MODIFICATIONS	
•Birth weight is associated with impaired endothelial function in childhood and adulthood •Prenatal growth restraint results in cardiovascular remodeling, and this may intensify the predisposition for future cardiovascular disease •Aortic and carotid IMT are increased in SGA children and adults •Excessive weight gain between 3 and 6 years of age is an independent predictor of carotid IMT •Obesity-related increase of carotid IMT has been reported since childhood and adolescence, and it persists in adulthood if obesity is not corrected on time •Higher IMT is associated also with preterm birth	
•Dyslipidemia has an important impact on cardio-metabolic health •Being born SGA is related to an unfavorable lipid profile in childhood and adolescence •Poor catch-up growth in height is associated with high levels of total cholesterol in adolescence •LBW has been associated with onset of dyslipidemia also in adulthood •Preterm birth is associated with higher triglycerides levels in childhood and higher total cholesterol levels in adult life	

IMT, Intima-Media Thickenss; LBW, Low-Birth Weight; SGA, Small for Gestational Age.

Prenatal growth restraint has been reported to result in cardiovascular remodeling. This may intensify the predisposition for future cardiovascular disease (116). Aortic and carotid intima-media thickness (IMT), which are markers of preclinical atherosclerosis, were found increased in SGA subjects both in childhood and young adulthood (117, 118). In SGA children, increased carotid IMT has been detected already at the age of 3-6 years (119). In a population comprehensive of SGA and AGA children, excessive weight gain between 3 and 6 years of age turned out to be an independent predictor of carotid IMT (119, 120). Obesity-related increase of carotid IMT has been reported since childhood and adolescence, and it persists in adulthood if obesity is not corrected on time (121).

Higher IMT has been associated also with preterm birth (73). In addition, homocysteine and heart-type fatty acid-binding protein, well known markers of myocardial and vascular impairment in adults (122, 123), have been found altered in preterm fetuses with IUGR (124). However, the significance of these markers in apparently healthy SGA infants with catch-up growth is still controversial (125, 126).

Dyslipidemia has an important impact on cardio-metabolic health (127), and many authors have tried to understand the effect of birth weight on lipid levels in childhood. Some studies suggested a relationship between being born SGA and an unfavorable lipid profile both in childhood and adolescence (128, 129). Besides, poor catch-up growth in height has been associated with high levels of total cholesterol in adolescence (129). LBW has been associated with onset of dyslipidemia also in adulthood (14).

A recent work described an association between preterm birth and total cholesterol levels in adult life, but results were not differentiated between those born AGA and SGA (73).

Beyond the effects of BMI percentile, children born preterm in the fifth grade had significantly higher triglycerides levels than peers born at term (128).

## 8 Metabolic syndrome

A systematic review focusing on the relationship between LBW and rapid catch-up growth with metabolic syndrome displayed that, in the largest number of studies including SGA-born children, adolescents and adults, both LBW and catch-up growth were associated with some aspects of metabolic syndrome in subsequent life (11). However, it was unclear which one between LBW and catch-up growth played the dominant role in leading to metabolic syndrome (11, 130) (**Table 5**).

**Table 5:** Metabolic syndrome.

METABOLIC SYNDROME
•LBW and catch-up growth are associated with MS in later life •It is not clear if LBW or catch-up growth plays the dominant role in leading to MS •Children born SGA have an adverse cardio-metabolic profile (BMI, SBP, HOMA-IR, triglycerides and triglycerides:HDL ratio) with a worsening of this profile during adolescence •Children born very preterm have low HDL-cholesterol concentrations and elevated BP •Accelerated early weight gain may contribute to the development of obesity in children born preterm •In animal studies accelerated weight gain amplifies the risk of MS, even in absence of intrauterine growth restriction, and the effects of LBW on adult phenotype can be reversed by preventing postnatal acceleration in weight
*Progression of IR and overall cardiovascular risk from childhood to adolescence in SGA population→ Accelerated postnatal weight gain is a key independent risk factor for subsequent MS*

BMI, Body Mass Index; BP, Blood Pressure; HDL, High Density Lipoprotein; HOMA-IR, HOmeostatic Model Assessment - Insulin Resistance; IR, Insulin Resistance; LBW, Low-Birth Weight; MS, Metabolic Syndrome; SBP, Systolic Blood Pressure; SGA, Small for Gestational Age.

An adverse cardio-metabolic profile, defined on the basis of clinical and biochemical parameters (BMI; systolic blood pressure; HOMA-IR; triglycerides and triglycerides:HDL ratio), was detected in children born SGA compared to AGA peers, with a worsening of this profile during adolescence. These data suggest a progression of insulin resistance and the overall cardiovascular risk from childhood to adolescence in SGA population (131).

A recent study compared metabolic syndrome parameters between very preterm and term children who were born AGA (41). The main finding was that some parameters were altered already in prepubertal age: namely, the proportion of children with low HDL-cholesterol concentrations and elevated blood pressure was higher in the very preterm group. A similar distribution of overweight and obesity was observed in both very preterm and term children. It is important to notice that, even though the two groups had similar BMIs, a stronger association with metabolic syndrome parameters was found in infants born very preterm (41). Accelerated weight gain during the first years of life may be a critical contributor to the development of obesity later in life in children born preterm (132). In another study involving adults, blood pressure was shown to be altered in 57.5% of those born very preterm, and elevated blood pressure was the most prevalent parameter of metabolic syndrome in this group. In addition, the mean value of HDL-cholesterol was significantly lower in adults born very preterm than in peers born at term (133).

Animal studies showed that accelerated weight gain amplifies the risk of metabolic disease, even in the absence of intrauterine growth restriction, and the effects of LBW on adult phenotype can be reversed with prevention of postnatal acceleration in weight. These observations support the concept that accelerated postnatal weight gain is a key independent risk factor for subsequent metabolic disease (26, 134).

## 9 Clinical management

Recently, an international consensus guideline clarified the main aspects and indications for SGA-born patients management from infancy to early adulthood. In this document, the authors stress out that children born SGA should be monitored during their first years of life, initially by a neonatologist and afterwards by a pediatrician to carefully evaluate their growth and weight gain (26). Namely, it is crucial to prevent excessive postnatal weight gain given its association with a less favorable cardio-metabolic profile in adult life. To do so, the authors recommend to avoid additional nutrition for healthy infants in the first 6 months of life, unless they endure malnutrition (26) (**Table 6**).

**Table 6:** Clinical management recommendations.

CLINICAL MANAGEMENT RECOMMENDATIONS
1. Evaluate weight, length, head circumference, and BMI every 3 months in the first year, every 6 months in the second year, and once a year thereafter 2. Plot growth parameters on appropriate growth chart 3. Breastfeeding is the ideal nutrition 4. Avoid additional nutrition for healthy infants in the first 6 months, unless malnourished 5. Recommend age-appropriate balanced diet 6. Infants born very preterm, severely SGA or with a syndrome causing growth impairment should be monitored more carefully and early referred to a pediatric endocrinologist 7. Avoid excessive weight gain in school years, recommending healthy lifestyle (balanced diet + regular physical activity) 8. Evaluate pubertal onset and progression 9. Evaluate metabolic parameters (FPG, OGTT, lipid profile) in children with overweight or obesity, family history of T2D, or clinical signs suggestive of metabolic disease

BMI, Body Mass Index; FPG, Fasting Plasma Glucose; OGTT, Oral Glucose Tolerance Test; SGA, Small for Gestational Age; T2D, Type-2 Diabetes.

Furthermore, in these guidelines a few high-risk populations are pointed out: very preterm or severely SGA-born children (birth weight and/or length < -3 SDs for gestational age), infants with complicated perinatal period, small head circumference, or with a syndrome causing growth impairment. These groups of children should be monitored more carefully, with shorter time intervals and early referred to a pediatric endocrinologist (26).

In the first two years of life, clinical management should focus on optimal nutrition to guarantee correct catch-up growth and to prevent excessive weight gain, as well as to exclude a possible genetic cause (26, 135). Recent works involving SGA-born children with a long-term follow-up agreed on the importance of breastfeeding in promoting adequate growth without causing unfavorable body composition or impaired insulin sensitivity and represent, therefore, the ideal nutrition for infants born SGA (26, 136, 137). Age-appropriate balanced diet containing macro- and micronutrients is recommended for optimal growth (26).

Early growth pattern evaluation is a crucial tool in the clinical management of these newborn and children. Weight, length, head circumference, and BMI should be monitored every 3 months during the first year of life, every 6 months in the second year of life, and afterwards once a year until the achievement of genetic target height. Growth parameters should be plotted on an appropriate growth chart and the obtained curve should be assessed (26).

In the following years of life, other than nutrition and growth, clinical management also focuses on pubertal onset and progression, and metabolic profile (26, 29). Metabolic parameters evaluation (fasting plasma glucose, oral glucose tolerance test, lipid profile) is not routinely indicated, but should be performed in children with overweight or obesity, family history of T2D, or clinical signs suggestive of a metabolic disease (26). Excessive weight gain (change in weight-for-length > 0.67 SDs), particularly in early childhood and during school years, should be avoided (35). It is essential to recommend a healthy lifestyle, comprehensive of a balanced diet and regular physical activity (26).

## 10 Supplementary considerations for economically developing nations

In developing nations approximately up to 50-60% of infants born SGA show satisfying catch-up growth in height and/or weight, and in these situations of poor nutrition and low protein dietary intake catch-up growth may be delayed up to the age of 5 years (138, 139).

Many factors may be involved in this negative outcome, primarily poor maternal nutrition, insufficient hygiene, infections, and low socioeconomic status (138). Therefore, in order to reduce SGA births in these countries and obtain a sufficient catch-up growth, it is crucial to improve nutrition, hygiene, and antenatal care during pregnancy, as well as to exclude subclinical hypothyroidism and to prevent and treat malaria where it is endemic (26).

Local reference growth charts should be used to monitor children born SGA (140). Given that many healthy children are below the 3^rd^ percentile for both height and weight considering WHO charts and that this phenomenon is emphasized in SGA, clinician should focus on the whole growth trajectory rather than on a single height and/or weight evaluation (26, 140).

Therefore, close monitoring of the growth trajectory is mandatory to prevent on one hand failure to thrive and on the other excessive catch-up in weight. Breastfeeding has been related with less rapid catch-up and growth lower fasting insulin and glucose concentrations both in infancy and childhood (141), with a protective effect against later obesity in numerous studies (142–144). Hence, WHO recommends exclusive breastfeeding or standard formulas rather than nutrient-enriched formulas during the first 6 months of life. Nevertheless, in LBW infants living in extremely poor regions, the priority is the avoidance of malnutrition and failure to thrive (145).

## 11 Conclusions

This review highlights how alterations in intrauterine growth have been associated with long-term cardiovascular and metabolic consequences. Children born SGA, particularly if they have experienced accelerated catch-up in weight in early life and/or obesity in later life, are at risk for insulin resistance and central adiposity since early childhood (51, 146, 147). They are also at risk for cardiovascular dysfunctions in later life (146). Additionally, growth trajectory in the first period of life is important in establishing risk for cardio-metabolic health across the lifespan (148). Thus, a careful observation of the children’s growth pattern, since the intrauterine period and the first years of life, should be emphasized, together with appropriate family counseling (22, 149). Notably, a healthier lifestyle for women in their fertility age, primarily while pregnant, is highly important to promote the offspring’s future health and wellbeing (11, 12), with a special focus on the first thousand days of life (19, 20).

## Author contributions

CC and VC: wrote the manuscript and were involved in literature search and drafting the paper. SV and FC: coordinated and approved the final version of the manuscript. The content has not been published or submitted for publication elsewhere.

## References

[1] Lozano R Naghavi M Foreman K Lim S Shibuya K Aboyans V. Global and regional mortality from 235 causes of death for 20 age groups in 1990 and 2010: a systematic analysis for the global burden of disease study 2010. Lancet (2012) 380(9859):2095–128. doi: 10.1016/S0140-6736(12)61728-0 PMC10790329 23245604

[2] Singhal A Lucas A. Early origins of cardiovascular disease: is there a unifying hypothesis? Lancet (2004) 363:1642–5. doi: 10.1016/S0140-6736(04)16210-7 15145640

[3] Gluckman PD Hanson MA. The developmental origins of the metabolic syndrome. Trends Endocrinol Metab (2004) 15:183–7. doi: 10.1016/j.tem.2004.03.002 15109618

[4] Phillips DIW. Endocrine programming and fetal origins of adult disease. Trends Endocrinol Metab (2002) 13:363. doi: 10.1016/S1043-2760(02)00696-3 12367815

[5] Kanaka-Gantenbein C Mastorakos G Chrousos GP. Endocrine-related causes and consequences of intrauterine growth retardation. In: Ann New York Acad Sci (2003) 997:150–7. doi: 10.1196/annals.1290.017 14644821

[6] Barker DJP Osmond C Golding J Kuh D Wadsworth MEJ. Growth *in utero*, blood pressure in childhood and adult life, and mortality from cardiovascular disease. Br Med J (1989) 298(6673):564–7. doi: 10.1136/bmj.298.6673.564 PMC1835925 2495113

[7] Saenger P Czernichow P Hughes I Reiter EO. Small for gestational age: short stature and beyond. Endocrine Rev (2007) 28:219–51. doi: 10.1210/er.2006-0039 17322454

[8] Huxley RR Shiell AW Law CM. The role of size at birth and postnatal catch-up growth in determining systolic blood pressure: a systematic review of the literature. J Hypertens (2000) 18(7):815–31. doi: 10.1097/00004872-200018070-00002 10930178

[9] Sunil TS Flores M Garcia GE. New evidence on the effects of international migration on the risk of low birthweight in Mexico. Matern Child Nutr (2012) 8(2):185–98. doi: 10.1111/j.1740-8709.2010.00277.x PMC6860498 20874845

[10] Bergmann RL Bergmann KE Dudenhausen JW. Undernutrition and growth restriction in pregnancy. Nestle Nutr Workshop Ser Pediatr Program (2008) 61:103–21. doi: 10.1159/000113181 18196948

[11] Kelishadi R Haghdoost AA Jamshidi F Aliramezany M Moosazadeh M. Low birthweight or rapid catch-up growth: which is more associated with cardiovascular disease and its risk factors in later life? a systematic review and cryptanalysis. Paediatrics Int Child Health (2015) 35:110–23. doi: 10.1179/2046905514Y.0000000136 25034799

[12] Kelishadi R Poursafa P. A review on the genetic, environmental, and lifestyle aspects of the early-life origins of cardiovascular disease. Curr Problems Pediatr Adolesc Health Care (2014) 44:54–72. doi: 10.1016/j.cppeds.2013.12.005 24607261

[13] García H Loureiro C Poggi H D’Apremont I Moore R Ossa JT. Insulin resistance parameters in children born very preterm and adequate for gestational age. Endocrinol Diabetes Metab (2022) 5(3):e00329. doi: 10.1002/edm2.329 35194980 PMC9094455

[14] Barker DJP Hales CN Fall CHD Osmond C Phipps K Clark PMS. Type 2 (non-insulin-dependent) diabetes mellitus, hypertension and hyperlipidaemia (syndrome x): relation to reduced fetal growth. Diabetologia (1993) 36(1):62–7. doi: 10.1007/BF00399095 8436255

[15] Barker DJP Osmond C. Infant mortality, childhood nutrition, and ischaemic heart disease in England and Wales. Lancet (1986) 327(8489):1077–81. doi: 10.1016/S0140-6736(86)91340-1 2871345

[16] Barker DJP Osmond C Forsén TJ Kajantie E Eriksson JG. Trajectories of growth among children who have coronary events as adults. N Engl J Med (2005) 353(17):1802–9. doi: 10.1056/NEJMoa044160 16251536

[17] Ekelund U Ong KK Linné Y Neovius M Brage S Dunger DB. Association of weight gain in infancy and early childhood with metabolic risk in young adults. J Clin Endocrinol Metab (2007) 92(1):98–103. doi: 10.1210/jc.2006-1071 17032722

[18] Skilton MR Marks GB Ayer JG Garden FL Garnett SP Harmer JA. Weight gain in infancy and vascular risk factors in later childhood. Pediatrics (2013) 131(6):e1821-8. doi: 10.1542/peds.2012-2789 23713097

[19] McCormick DP Sarpong K Jordan L Ray LA Jain S. Infant obesity: are we ready to make this diagnosis? J Pediatr (2010) 157(1):15–9. doi: 10.1016/j.jpeds.2010.01.028 20338575

[20] Lurbe E Aguilar F Álvarez J Redon P Torró MI Redon J. Determinants of cardiometabolic risk factors in the first decade of life. Hypertension (2018) 71(3):437–43. doi: 10.1161/HYPERTENSIONAHA.117.10529 29358459

[21] Weihe P Weihrauch-Blüher S. Metabolic syndrome in children and adolescents: diagnostic criteria, therapeutic options and perspectives. Curr Obes Rep (2019) 8:472–9. doi: 10.1007/s13679-019-00357-x 31691175

[22] Nordman H Jääskeläinen J Voutilainen R. Birth size as a determinant of cardiometabolic risk factors in children. Hormone Res Paediatrics (2020) 93:144–53. doi: 10.1159/000509932 32846418

[23] Campbell MK Cartier S Xie B Kouniakis G Huang W Han V. Determinants of small for gestational age birth at term. Paediatr Perinat Epidemiol (2012) 26(6):525–33. doi: 10.1111/j.1365-3016.2012.01319.x 23061688

[24] Finken MJJ van der Steen M Smeets CCJ Walenkamp MJE De Bruin C Hokken-Koelega ACS. Children born small for gestational age: differential diagnosis, molecular genetic evaluation, and implications. Endocrine Rev (2018) 39:851–94. doi: 10.1210/er.2018-00083 29982551

[25] Mandruzzato G Antsaklis A Botet F Chervenak FA Figueras F Grunebaum A. Intrauterine restriction (IUGR). J Perinatal Med (2008) 36:277–81. doi: 10.1515/JPM.2008.050 18598115

[26] Hokken-Koelega ACS van der Steen M Boguszewski MCS Cianfarani S Dahlgren J Horikawa R. International consensus guideline on small for gestational age: etiology and management from infancy to early adulthood. Endocr Rev (2023) 44(3):539–65. doi: 10.1210/endrev/bnad002 36635911 PMC10166266

[27] Lee PA Chernausek SD Hokken-koelega ACS. International small for gestational age advisory board consensus. Pediatrics (2003) 111(6):1253–61. doi: 10.1542/peds.111.6.1253 12777538

[28] Chauhan SP Rice MM Grobman WA Bailit J Reddy UM Wapner RJ. Neonatal morbidity of small- and Large-for-Gestational-Age neonates born at term in uncomplicated pregnancies. Obstet Gynecol (2017) 130(3):511–9. doi: 10.1097/AOG.0000000000002199 PMC5578445 28796674

[29] Clayton PE Cianfarani S Czernichow P Johannsson G Rapaport R Rogol AD. Consensus statement: management of the child born small for gestational age through to adulthood: a consensus statement of the international societies of pediatric endocrinology and the growth hormone research society. J Clin Endocrinol Metab (2007) 92(3):804–10. doi: 10.1210/jc.2006-2017 17200164

[30] Albertsson-Wikland K Karlberg J. Natural growth in children born SGA with and without catch up growth. Horm Res (2003) 59(SUPPL. 1):129. doi: 10.1159/000067839 12638524

[31] Who: recommended definitions, terminology and format for statistical tables related to the perinatal period and use of a new certificate for cause of perinatal deaths. Acta Obstet Gynecol Scand (1977) 56(3):247–53. 560099

[32] Walani SR. Global burden of preterm birth. Int J Gynecol Obstet (2020) 150(1):31–3. doi: 10.1002/ijgo.13195 32524596

[33] Howson CP Kinney M McDougall L Lawn JE. Born too soon: the global action report on preterm birth. Geneva: World Health Organization; 2012. Reprod Health (2013) 10(Suppl1):S1. doi: 10.1186/1742-4755-10-S1-S1

[34] Vogel JP Chawanpaiboon S Moller AB Watananirun K Bonet M Lumbiganon P. The global epidemiology of preterm birth. Best Pract Res: Clin Obstetrics Gynaecol (2018) 52:3–12. doi: 10.1016/j.bpobgyn.2018.04.003 29779863

[35] Ong KK. Catch-up growth in small for gestational age babies: good or bad? Curr Opin Endocrinol Diabetes Obes (2007) 14:30–4. doi: 10.1097/MED.0b013e328013da6c 17940416

[36] Ong K Loos R. Rapid infancy weight gain and subsequent obesity: systematic reviews and hopeful suggestions. Acta Paediatrica Int J Paediatrics (2006) 95:904–8. doi: 10.1080/08035250600719754 16882560

[37] Gluckman PD Hanson MA Cooper C Thornburg KL. Effect of *In utero* and early-life conditions on adult health and disease. N Engl J Med (2008) 359(1):61–73. doi: 10.1056/NEJMra0708473 18596274 PMC3923653

[38] Gamborg M Andersen PK Baker JL Budtz-Jørgensen E Jørgensen T Jensen G. Life course path analysis of birth weight, childhood growth, and adult systolic blood pressure. Am J Epidemiol (2009) 169(10):1167–78. doi: 10.1093/aje/kwp047 PMC2732973 19357327

[39] Fujita Y Kouda K Nakamura H Iki M. Association of rapid weight gain during early childhood with cardiovascular risk factors in Japanese adolescents. J Epidemiol (2013) 23(2):103–8. doi: 10.2188/jea.JE20120107 PMC3700244 23269125

[40] WHO. WHO. world health organization (WHO): obesity and overweight. World Health Organization (2020). Available at: https://www.who.int/news-room/fact-sheets/detail/obesity-and-overweight.

[41] Cook S Weitzman M Auinger P Nguyen M Dietz WH. Prevalence of a metabolic syndrome phenotype in adolescents. Arch Pediatr Adolesc Med (2003) 157(8):821. doi: 10.1001/archpedi.157.8.821 12912790

[42] Ahrens W Moreno L Mårild S Molnár D Siani A De Henauw S. Metabolic syndrome in young children: definitions and results of the IDEFICS study. Int J Obes (2014) 38:S4–14. doi: 10.1038/ijo.2014.130 25376220

[43] Chiarelli F Mohn A. Early diagnosis of metabolic syndrome in children. Lancet Child Adolesc Heal (2017) 1(2):86–8. doi: 10.1016/S2352-4642(17)30043-3 30169210

[44] Barker DJP. The fetal and infant origins of adult disease. Br Med J (1990) 301:1111. doi: 10.1136/bmj.301.6761.1111 2252919 PMC1664286

[45] Newsome CA Shiell AW Fall CHD Phillips DIW Shier R Law CM. Is birth weight related to later glucose and insulin metabolism? - a systematic review. Diabetic Med (2003) 20:339–48. doi: 10.1046/j.1464-5491.2003.00871.x 12752481

[46] Hattersley AT Tooke JE. The fetal insulin hypothesis: an alternative explanation of the association of low birthweight with diabetes and vascular disease. Lancet (1999) 353:1789–92. doi: 10.1016/S0140-6736(98)07546-1 10348008

[47] Hattersley AT Turner RC Patel P O’Rahilly S Hattersley AT Patel P. Linkage of type 2 diabetes to the glucokinase gene. Lancet (1992) 339(8805):1307–10. doi: 10.1016/0140-6736(92)91958-B 1349989

[48] Jones RH Ozanne SE. Fetal programming of glucose-insulin metabolism. Mol Cell Endocrinol (2009) 297(1–2):4–9. doi: 10.1016/j.mce.2008.06.020 18662742

[49] Hales CN Barker DJP Clark PMS Cox LJ Fall C Osmond C. Fetal and infant growth and impaired glucose tolerance at age 64. Br Med J (1991) 303(6809):1019–22. doi: 10.1136/bmj.303.6809.1019 PMC1671766 1954451

[50] Druet C Ong KK. Early childhood predictors of adult body composition. Best Pract Res: Clin Endocrinol Metab (2008) 22:489–502. doi: 10.1016/j.beem.2008.02.002 18538288

[51] Ibáñez L Ong K Dunger DB De Zegher F. Early development of adiposity and insulin resistance after catch-up weight gain in small-for-gestational-age children. J Clin Endocrinol Metab (2006) 91(6):2153–8. doi: 10.1210/jc.2005-2778. 16537681

[52] Jaquet D Deghmoun S Chevenne D Collin D Czernichow P Lévy-Marchal C. Dynamic change in adiposity from fetal to postnatal life is involved in the metabolic syndrome associated with reduced fetal growth. Diabetologia (2005) 48(5):849–55. doi: 10.1007/s00125-005-1724-4 15834547

[53] Desai M Jellyman JK Ross MG. Epigenomics, gestational programming and risk of metabolic syndrome. Int J Obes (2015) 39:633–41. doi: 10.1038/ijo.2015.13 25640766

[54] Costantino S Mohammed SA Ambrosini S Paneni F. Epigenetic processing in cardiometabolic disease. Atherosclerosis (2019) 281:150–8. doi: 10.1016/j.atherosclerosis.2018.09.029 30290963

[55] Gonzalez-Nahm S Mendez MA Benjamin-Neelon SE Murphy SK Hogan VK Rowley DL. DNA Methylation of imprinted genes at birth is associated with child weight status at birth, 1 year, and 3 years. Clin Epigenet [Internet] (2018) 10(1):90. doi: 10.1186/s13148-018-0521-0 PMC6025828 29988473

[56] Fradin D Boëlle PY Belot MP Lachaux F Tost J Besse C. Genome-wide methylation analysis identifies specific epigenetic marks in severely obese children. Sci Rep (2017) 7:46311. doi: 10.1038/srep46311 28387357 PMC5384222

[57] Agha G Hajj H Rifas-Shiman SL Just AC Hivert MF Burris HH. Birth weight-for-gestational age is associated with DNA methylation at birth and in childhood. Clin Epigenetics (2016) 8(1):118. doi: 10.1186/s13148-016-0285-3 27891191 PMC5112715

[58] Whincup PH Kaye SJ Owen CG Huxley R Cook DG Anazawa S. Birth weight and risk of type 2 diabetes a systematic review. JAMA (2008) 300:2886–97. doi: 10.1001/jama.2008.886 19109117

[59] Wei JN Sung FC Li CY Chang CH Lin RS Lin CC. Low birth weight and high birth weight infants are both at an increased risk to have type 2 diabetes among schoolchildren in Taiwan. Diabetes Care (2003) 26(2):343–8. doi: 10.2337/diacare.26.2.343 12547860

[60] Freedman DS Srinivasan SR Burke GL Shear CL Smoak CG Harsha DW. Relation of body fat distribution to hyperinsulinemia in children and adolescents: the bogalusa heart study. Am J Clin Nutr (1987) 46(3):403–10. doi: 10.1093/ajcn/46.3.403 3307372

[61] Whincup PH Cook DG Adshead F Taylor SJC Walker M Papacosta O. Childhood size is more strongly related than size at birth to glucose and insulin levels in 10-11-year-old children. Diabetologia (1997) 40(3):319–26. doi: 10.1007/s001250050681 9084971

[62] Arends NJT Boonstra VH Duivenvoorden HJ Hofman PL Cutfield WS Hokken-Koelega ACS. Reduced insulin sensitivity and the presence of cardiovascular risk factors in short prepubertal children born small for gestational age (SGA). Clin Endocrinol (Oxf) (2005) 62(1):44–50. doi: 10.1111/j.1365-2265.2004.02171.x 15638869

[63] Brufani C Grossi A Fintini D Tozzi A Nocerino V Patera PI. Obese children with low birth weight demonstrate impaired β-cell function during oral glucose tolerance test. J Clin Endocrinol Metab (2009) 94(11):4448–52. doi: 10.1210/jc.2009-1079 19820011

[64] Sancakli O Darendeliler F Bas F Gokcay G Disci R Aki S. Insulin, adiponectin, IGFBP-1 levels and body composition in small for gestational age born non-obese children during prepubertal ages. Clin Endocrinol (Oxf) (2008) 69(1):88–92. doi: 10.1111/j.1365-2265.2007.03138.x 18031314

[65] Sinaiko AR Donahue RP Jacobs DR Prineas RJ. Relation of weight and rate of increase in weight during childhood and adolescence to body size, blood pressure, fasting insulin, and lipids in young adults: the minneapolis children’s blood pressure study. Circulation (1999) 99(11):1471–6. doi: 10.1161/01.CIR.99.11.1471 10086972

[66] Eriksson JG Forsén T Tuomilehto J Osmond C Barker DJP. Early adiposity rebound in childhood and risk of type 2 diabetes in adult life. Diabetologia (2003) 46(2):190–4. doi: 10.1007/s00125-002-1012-5 12627317

[67] Ong KK Petry CJ Emmett PM Saradhu MS Kiess W Hales CN. Insulin sensitivity and secretion in normal children related to size at birth, postnatal growth, and plasma insulin-like growth factor-I levels. Diabetologia (2004) 47(6):1064–70. doi: 10.1007/s00125-004-1405-8 15156313

[68] Mericq V Ong KK Bazaes R Peña V Avila A Salazar T. Longitudinal changes in insulin sensitivity and secretion from birth to age three years in small- and appropriate-for-gestational-age children. Diabetologia (2005) 48(12):2609–14. doi: 10.1007/s00125-005-0036-z 16283238

[69] Finken MJJ Keijzer-Veen MG Dekker FW Frölich M Hille ETM Romijn JA. Preterm birth and later insulin resistance: effects of birth weight and postnatal growth in a population based longitudinal study from birth into adult life. Diabetologia (2006) 49(3):478–85. doi: 10.1007/s00125-005-0118-y 16450090

[70] Euser AM Finken MJJ Keijzer-Veen MG Hille ETM Wit JM Dekker FW. Associations between prenatal and infancy weight gain and BMI, fat mass, and fat distribution in young adulthood: a prospective cohort study in males and females born very preterm. Am J Clin Nutr (2005) 81(2):480–7. doi: 10.1093/ajcn.81.2.480 15699238

[71] Hofman PL Regan F Jackson WE Jefferies C Knight DB Robinson EM. Premature birth and later insulin resistance. N Engl J Med (2004) 351(21):2179–86. doi: 10.1056/NEJMoa042275 15548778

[72] Bazaes RA Alegría A Pittaluga E Ávila A Íñiguez G Mericq V. Determinants of insulin sensitivity and secretion in very-low-birth-weight children. J Clin Endocrinol Metab (2004) 89(3):1267–72. doi: 10.1210/jc.2003-031239 15001621

[73] Markopoulou P Papanikolaou E Analytis A Zoumakis E Siahanidou T. Preterm birth as a risk factor for metabolic syndrome and cardiovascular disease in adult life: a systematic review and meta-analysis. J Pediatr (2019) 210:69–80.e5. doi: 10.1016/j.jpeds.2019.02.041 30992219

[74] Niklasson A Engström E Hård AL Wikland KA Hellström A. Growth in very preterm children: a longitudinal study. Pediatr Res (2003) 54(6):899–905. doi: 10.1203/01.PDR.0000091287.38691.EF 12930904

[75] Knops NBB Sneeuw KCA Brand R Hille ETM den Ouden AL Wit JM. Catch-up growth up to ten years of age in children born very preterm or with very low birth weight. BMC Pediatr (2005) 5:26. doi: 10.1186/1471-2431-5-26 16033642 PMC1199602

[76] Singhal A Fewtrell M Cole TJ Lucas A. Low nutrient intake and early growth for later insulin resistance in adolescents born preterm. Lancet (2003) 361(9363):1089–97. doi: 10.1016/S0140-6736(03)12895-4 12672313

[77] Matthews DR Hosker JP Rudenski AS Naylor BA Treacher DF Turner RC. Homeostasis model assessment: insulin resistance and beta-cell function from fasting plasma glucose and insulin concentrations in man. Diabetologia (1985) 28(7):412–9. doi: 10.1007/BF00280883 3899825

[78] Huang YT Lin HY Wang CH Su BH Lin CC. Association of preterm birth and small for gestational age with metabolic outcomes in children and adolescents: a population-based cohort study from Taiwan. Pediatr Neonatol [Internet] (2018) 59(2):147–53. doi: 10.1016/j.pedneo.2017.07.007 28789832

[79] Crump C Sundquist J Sundquist K. Preterm birth and risk of type 1 and type 2 diabetes: a national cohort study. Diabetologia (2020) 63(3):508–18. doi: 10.1007/s00125-019-05044-z PMC6997251 31802143

[80] Whitaker RC Wright JA Pepe MS Seidel KD Dietz WH. Predicting obesity in young adulthood from childhood and parental obesity. N Engl J Med (1997) 337(13):869-73. doi: 10.1056/NEJM199709253371301 9302300

[81] Lindell N Bladh M Carlsson A Josefsson A Aakesson K Samuelsson U. Size for gestational age affects the risk for type 1 diabetes in children and adolescents: a Swedish national case–control study. Diabetologia (2021) 64(5):1113–20. doi: 10.1007/s00125-021-05381-y PMC8012313 33544169

[82] Prinz N Putri RR Reinehr T Danielsson P Weghuber D Norman M. The association between perinatal factors and cardiometabolic risk factors in children and adolescents with overweight or obesity: a retrospective two-cohort study. PloS Med (2023) 20(1):1–17. doi: 10.1371/journal.pmed.1004165 PMC9886302 36638094

[83] Monteiro POA Victora CG. Rapid growth in infancy and childhood and obesity in later life - a systematic review. Obes Rev (2005) 6:143–54. doi: 10.1111/j.1467-789X.2005.00183.x 15836465

[84] Ibáñez L Lopez-Bermejo A Díaz M Suárez L De Zegher F. Low-birth weight children develop lower sex hormone binding globulin and higher dehydroepiandrosterone sulfate levels and aggravate their visceral adiposity and hypoadiponectinemia between six and eight years of age. J Clin Endocrinol Metab (2009) 94(10):3696–9. doi: 10.1210/jc.2009-0789. 19737922

[85] Leunissen RWJ Stijnen T Hokken-Koelega ACS. Influence of birth size on body composition in early adulthood: the programming factors for growth and metabolism (PROGRAM)-study. Clin Endocrinol (Oxf) (2009) 70(2):245–51. doi: 10.1111/j.1365-2265.2008.03320.x 18616715

[86] Ibáñez L López-Bermejo A Díaz M Marcos MV Casano P De Zegher F. Abdominal fat partitioning and high-molecular-weight adiponectin in short children born small for gestational age. J Clin Endocrinol Metab (2009) 94(3):1049–52. doi: 10.1210/jc.2008-2176. 19066297

[87] Singh P Covassin N Marlatt K Gadde KM Heymsfield SB. Obesity, body composition, and sex hormones: implications for cardiovascular risk. Compr Physiol (2022) 12(1):2949–93. doi: 10.1002/cphy.c210014 PMC10068688 34964120

[88] Neeland IJ Poirier P Després J-P. Cardiovascular and metabolic heterogeneity of obesity. Circulation (2018) 137(13):1391–406. doi: 10.1161/CIRCULATIONAHA.117.029617 PMC5875734 29581366

[89] Maffeis C Morandi A. Body composition and insulin resistance in children. Eur J Clin Nutr (2018) 72(9):1239–45. doi: 10.1038/s41430-018-0239-2 30185840

[90] Caprio S Hyman LD Limb C Mccarthy S Lange R Sherwin RS. Central adiposity adolescent girls and its metabolic correlates in obese. Am J Physiol (2018) 269(1 Pt 1):E118–26. doi: 10.1152/ajpendo.1995.269.1.E118 7631766

[91] Gower BA Nagy TR Trowbridge CA Dezenberg C Goran MI. Fat distribution and insulin response in prepubertal African American and white children. Am J Clin Nutr (1998) 67(5):821–7. doi: 10.1093/ajcn/67.5.821 9583837

[92] Bacha F Saad R Gungor N Janosky J Arslanian SA. Obesity, regional fat distribution, and syndrome X in obese black versus white adolescents: race differential in diabetogenic and atherogenic risk factors. J Clin Endocrinol Metab (2003) 88(6):2534–40. doi: 10.1210/jc.2002-021267 12788850

[93] Bacha F Saad R Gungor N Arslanian SA. Are obesity-related metabolic risk factors modulated by the degree of insulin resistance in adolescents? Diabetes Care (2006) 29(7):1599–604. doi: 10.2337/dc06-0581 16801585

[94] Misra A Vikram NK Arya S Pandey RM Dhingra V Chatterjee A. High prevalence of insulin resistance in postpubertal Asian Indian children is associated with adverse truncal body fat patterning, abdominal adiposity and excess body fat. Int J Obes (2004) 28(10):1217–26. doi: 10.1038/sj.ijo.0802704 15314636

[95] Weiss R Dufour S Taksali SE Tamborlane WV Petersen KF Bonadonna RC. Prediabetes in obese youth: a syndrome of impaired glucose tolerance, severe insulin resistance, and altered myocellular and abdominal fat partitioning. Lancet (2003) 362(9388):951–7. doi: 10.1016/S0140-6736(03)14364-4 PMC2995523 14511928

[96] Taal HR Vd Heijden AJ Steegers EAP Hofman A Jaddoe VWV. Small and large size for gestational age at birth, infant growth, and childhood overweight. Obesity (2013) 21(6):1261–8. doi: 10.1002/oby.20116 23666877

[97] Tobisch B Blatniczky L Barkai L. Cardiometabolic risk factors and insulin resistance in obese children and adolescents: relation to puberty. Pediatr Obes (2015) 10(1):37–44. doi: 10.1111/j.2047-6310.2013.00202.x 24227418

[98] Prince RL Kuk JL Ambler KA Dhaliwal J Ball GDC. Predictors of metabolically healthy obesity in children. Diabetes Care (2014) 37(5):1462–8. doi: 10.2337/dc13-1697 24574347

[99] Tam CHT Wang Y Luan J Lee HM Luk AOY Tutino GE. Non-linear relationship between birthweight and cardiometabolic risk factors in Chinese adolescents and adults. Diabetes Med (2015) 32(2):220–5. doi: 10.1111/dme.12630 25388749

[100] Embleton ND Korada M Wood CL Pearce MS Swamy R Cheetham TD. Catch-up growth and metabolic outcomes in adolescents born preterm. Arch Dis Child (2016) 101(11):1026–31. doi: 10.1136/archdischild-2015-310190 27288431

[101] Geserick M Vogel M Gausche R Lipek T Spielau U Keller E. Acceleration of BMI in early childhood and risk of sustained obesity. N Engl J Med (2018) 379(14):1303–12. doi: 10.1056/NEJMoa1803527 30281992

[102] Huxley R Neil A Collins R. Unravelling the fetal origins hypothesis: is there really an inverse association between birthweight and subsequent blood pressure? Lancet (2002) 360(9334):659–65. doi: 10.1016/S0140-6736(02)09834-3 12241871

[103] Chiolero A Paradis G Kaufman JS. Assessing the possible direct effect of birth weight on childhood blood pressure: a sensitivity analysis. Am J Epidemiol (2014) 179(1):4–11. doi: 10.1093/aje/kwt228 24186972

[104] Tenhola S Rahiala E Martikainen A Halonen P Voutilainen R. Blood pressure, serum lipids, fasting insulin, and adrenal hormones in 12-year-old children born with maternal preeclampsia. J Clin Endocrinol Metab (2003) 88(3):1217–22. doi: 10.1210/jc.2002-020903 12629109

[105] Tenhola S Rahiala E Halonen P Vanninen E Voutilainen R. Maternal preeclampsia predicts elevated blood pressure in 12-year-old children: evaluation by ambulatory blood pressure monitoring. Pediatr Res (2006) 59(2):320–4. doi: 10.1203/01.pdr.0000196734.54473.e3 16439600

[106] Lurbe E Garcia-Vicent C Torro I Fayos JL Aguilar F De Llano JM. First-year blood pressure increase steepest in low birthweight newborns. J Hypertens (2007) 25(1):81–6. doi: 10.1097/HJH.0b013e32801040ec 17143177

[107] Mzayek F Cruickshank JK Amoah D Srinivasan S Chen W Berenson GS. Birth weight was longitudinally associated with cardiometabolic risk markers in mid-adulthood. Ann Epidemiol (2016) 26(9):643–7. doi: 10.1016/j.annepidem.2016.07.013 PMC5115831 27664850

[108] Bowers K Liu G Wang P Ye T Tian Z Liu E. Birth weight, postnatal weight change, and risk for high blood pressure among Chinese children. Pediatrics (2011) 127(5):e1272-9. doi: 10.1542/peds.2010-2213 21502227 PMC3387869

[109] Liang Y Hou D Shan X Zhao X Hu Y Jiang B. Cardiovascular remodeling relates to elevated childhood blood pressure: Beijing blood pressure cohort study. Int J Cardiol (2014) 177(3):836–9. doi: 10.1016/j.ijcard.2014.11.013 25465829

[110] Kelly RK Thomson R Smith KJ Dwyer T Venn A Magnussen CG. Factors affecting tracking of blood pressure from childhood to adulthood: the childhood determinants of adult health study. J Pediatr (2020) 167(6):1428.e2. doi: 10.1016/j.jpeds.2015.07.055 26342719

[111] Zetterström K Lindeberg S Haglund B Magnuson A Hanson U. Being born small for gestational age increases the risk of severe pre-eclampsia. BJOG Int J Obstet Gynaecol (2007) 114(3):319–24. doi: 10.1111/j.1471-0528.2006.01231.x 17261123

[112] Rodriguez J Adams-Chapman I Affuso O Azuero A Downs CA Turner-Henson A. Weight gain and blood pressure in toddlers born very preterm. Nurs Res (2020) 69(3):238–43. doi: 10.1097/NNR.0000000000000415 31934944

[113] Faienza MF Brunetti G Delvecchio M Zito A de Palma FD Cortese F. Vascular function and myocardial performance indices in children born small for gestational age. Circ J (2016) 80(4):958–63. doi: 10.1253/circj.CJ-15-1038 26861187

[114] Franco MCP Christofalo DMJ Sawaya AL Ajzen SA Sesso R. Effects of low birth weight in 8- to 13-year-old children: implications in endothelial function and uric acid levels. Hypertension (2006) 48(1):45–50. doi: 10.1161/01.HYP.0000223446.49596.3a 16682609

[115] Leeson CPM Kattenhorn M Morley R Lucas A Deanfield JE. Impact of low birth weight and cardiovascular risk factors on endothelial function in early adult life. Circulation (2001) 103(9):1264–8. doi: 10.1161/01.CIR.103.9.1264 11238271

[116] Crispi F Figueras F Cruz-Lemini M Bartrons J Bijnens B Gratacos E. Cardiovascular programming in children born small for gestational age and relationship with prenatal signs of severity. Am J Obstet Gynecol (2012) 207(2):121.e1–9. doi: 10.1016/j.ajog.2012.05.011 22717268

[117] Leunissen RWJ Kerkhof GF Stijnen T Hokken-Koelega ACS. Effect of birth size and catch-up growth on adult blood pressure and carotid intima-media thickness. Horm Res Paediatr (2012) 77(6):394–401. doi: 10.1159/000338791 22760117

[118] Cruz-Lemini M Crispi F Valenzuela-Alcaraz B Figueras F Sitges M Bijnens B. Fetal cardiovascular remodeling persists at 6 months in infants with intrauterine growth restriction. Ultrasound Obstet Gynecol (2016) 48(3):349–56. doi: 10.1002/uog.15767 26415719

[119] Sebastiani G García-Beltran C Pie S Guerra A López-Bermejo A de Toledo JS. The sequence of prenatal growth restraint and postnatal catch-up growth: normal heart but thicker intima-media and more pre-peritoneal fat in late infancy. Pediatr Obes (2019) 14(3):e12476. doi: 10.1111/ijpo.12476 30362284

[120] David D Chiavaroli V Lanci M Sabatini L Greco S Carinci S. Neonatal diagnosis of Marcus gunn jaw-winking syndrome. Clin Case Rep (2021) 9(2):866–9. doi: 10.1002/ccr3.3664 PMC7869362 33598261

[121] Juonala M Magnussen CG Berenson GS Venn A Burns TL Sabin MA. Childhood adiposity, adult adiposity, and cardiovascular risk factors. N Engl J Med (2011) 365(20):1876–85. doi: 10.1056/NEJMoa1010112 22087679

[122] Vizzardi E Bonadei I Zanini G Frattini S Fiorina C Raddino R. Homocysteine and heart failure: an overview. Recent Patents Cardiovasc Drug Discovery (2009) 4:15–21. doi: 10.2174/157489009787259991 19149701

[123] Azzazy HME Pelsers MMAL Christenson RH. Unbound free fatty acids and heart-type fatty acid-binding protein: diagnostic assays and clinical applications. Clin Chem (2006) 52:19–29. doi: 10.1373/clinchem.2005.056143 16269514

[124] Crispi F Hernandez-Andrade E Pelsers MMAL Plasencia W Benavides-Serralde JA Eixarch E. Cardiac dysfunction and cell damage across clinical stages of severity in growth-restricted fetuses. Am J Obstet Gynecol (2008) 199(3):254.e1–8. doi: 10.1016/j.ajog.2008.06.056 18771973

[125] Gomes TS Lindner U Tennekoon KH Karandagoda W Gortner L Obeid R. Homocysteine in small-for-gestational age and appropriate-for-gestational age preterm neonates from mothers receiving folic acid supplementation. Clin Chem Lab Med (2010) 48(8):1157–61. doi: 10.1515/CCLM.2010.235 20482301

[126] Perez-Cruz M Crispi F Fernández MT Parra JA Valls A Gomez Roig MD. Cord blood biomarkers of cardiac dysfunction and damage in term growth-restricted fetuses classified by severity criteria. Fetal Diagn Ther (2018) 44(4):271–6. doi: 10.1159/000484315 29190628

[127] Frohlich J Al-Sarraf A. Cardiovascular risk and atherosclerosis prevention. Cardiovasc Pathol (2013) 22:16–8. doi: 10.1016/j.carpath.2012.03.001 22502868

[128] Mullett MD Cottrell L Lilly C Gadikota K Dong L Hobbs G. Association between birth characteristics and coronary disease risk factors among fifth graders. J Pediatr (2014) 164(1):78–82. doi: 10.1016/j.jpeds.2013.08.064 24120018

[129] Tenhola S Martikainen A Rahiala E HerrgÅrd E Halonen P Voutilainen R. Serum lipid concentrations and growth characteristics in 12-year-old children born small for gestational age. Pediatr Res (2000) 48(5):623–8. doi: 10.1203/00006450-200011000-00012 11044482

[130] Nobili V Alisi A Panera N Agostoni C. Low birth weight and catch-up-growth associated with metabolic syndrome: a ten year systematic review. Pediatr Endocrinol Rev (2008) 6:241–7. 19202511

[131] Chiavaroli V Marcovecchio ML De Giorgis T Diesse L Chiarelli F Mohn A. Progression of cardio-metabolic risk factors in subjects born small and large for gestational age. PloS One (2014) 9(8):e104278. doi: 10.1371/journal.pone.0104278 25117750 PMC4130586

[132] Ou-Yang MC Sun Y Liebowitz M Chen CC Fang ML Dai W. Accelerated weight gain, prematurity, and the risk of childhood obesity: a meta-analysis and systematic review. PloS One (2020) 15:e0232238. doi: 10.1371/journal.pone.0232238 32369502 PMC7199955

[133] Heidemann LA Procianoy RS Silveira RC. Prevalence of metabolic syndrome-like in the follow-up of very low birth weight preterm infants and associated factors. J Pediatr (Rio J) (2019) 95(3):291–7. doi: 10.1016/j.jped.2018.02.009 29705050

[134] Leger J Levy-Marchal C Bloch J Pinet A Chevenne D Porquet D. Reduced final height and indications for insulin resistance in 20 year olds born small for gestational age: regional cohort study. Br Med J (1997) 315(7104):341–7. doi: 10.1136/bmj.315.7104.341 PMC2127259 9270455

[135] Katz J Lee ACC Kozuki N Lawn JE Cousens S Blencowe H. Mortality risk in preterm and small-for-gestational-age infants in low-income and middle-income countries: a pooled country analysis. Lancet (2013) 382(9890):417–25. doi: 10.1016/S0140-6736(13)60993-9 PMC3796350 23746775

[136] Santiago ACT da Cunha LPM Vieira NSA Oliveira Moreira LM de Oliveira PR Lyra PPR. Breastfeeding in children born small for gestational age and future nutritional and metabolic outcomes: a systematic review. J Pediatr (Rio J) (2019) 95(3):264–74. doi: 10.1016/j.jped.2018.06.013 30138579

[137] Hwang IT. Long-term care, from neonatal period to adulthood, of children born small for gestational age. Clin Pediatr Endocrinol (2019) 28(4):97–103. doi: 10.1297/cpe.28.97 31666762 PMC6801360

[138] Gurung S Tong HH Bryce E Katz J Lee AC Black RE. A systematic review on estimating population attributable fraction for risk factors for small-for-gestational-age births in 81 low and middle-income countries. J Glob Health (2022) 12:1–15. doi: 10.7189/jogh.12.04024 PMC8942297 35356650

[139] Khadilkar V Mandlik R Palande S Pandit D Chawla M Nadar R. Growth status of small for gestational age Indian children from two socioeconomic strata. Indian J Endocrinol Metab (2016) 20(4):531–5. doi: 10.4103/2230-8210.183473 PMC4911844 27366721

[140] Khadilkar V Khadilkar A Chiplonkar S. Growth performance of affluent Indian preschool children: a comparison with the new WHO growth standard. Indian Pediatr (2010) 317:2008–11. doi: 10.1007/s13312-010-0147-6 20308761

[141] Gupta M Zaheer Jora R Kaul V Kaul R. Breast feeding and insulin levels in low birth weight neonates: a randomized study. Indian J Pediatr (2010) 77(5):509–13. doi: 10.1007/s12098-010-0065-6 20401702

[142] Arenz S Rückerl R Koletzko B Von Kries R. Breast-feeding and childhood obesity - a systematic review. Int J Obes (2004) 28(10):1247–56. doi: 10.1038/sj.ijo.0802758 15314625

[143] Owen CG Martin RM Whincup PH Smith GD Cook DG. Effect of infant feeding on the risk of obesity across the life course: a quantitative review of published evidence. Pediatrics (2005) 115(5):1367–77. doi: 10.1542/peds.2004-1176 15867049

[144] Koletzko B Von Kries R Monasterolo RC Subías JE Scaglioni S Giovannini M. Can infant feeding choices modulate later obesity risk? Am J Clin Nutr (2009) 89(5):1502–9. doi: 10.3945/ajcn.2009.27113D 19321574

[145] Victora CG Barros FC Horta BL Martorell R. Short-term benefits of catch-up growth for small-for-gestational-age infants. Int J Epidemiol (2001) 30(6):1325–30. doi: 10.1093/ije/30.6.1325 11821340

[146] Laitinen T Laitinen TT Pahkala K Magnussen CG Viikari JSA Oikonen M. Ideal cardiovascular health in childhood and cardiometabolic outcomes in adulthood: the cardiovascular risk in young finns study. Circulation (2012) 125(16):1971–8. doi: 10.1161/CIRCULATIONAHA.111.073585 22452832

[147] Maguolo A Olivieri F Zusi C Miraglia Del Giudice E Morandi A Maffeis C. The risk of metabolic derangements is higher in children and adolescents with overweight or obesity born small for gestational age. Nutr Metab Cardiovasc Dis (2021) 31(6):1903–10. doi: 10.1016/j.numecd.2021.02.025 33941428

[148] Woo JG. Infant growth and long-term cardiometabolic health: a review of recent findings. Curr Nutr Rep (2019) 8:29–41. doi: 10.1007/s13668-019-0259-0 30729427

[149] Fogelholm M Nuutinen O Pasanen M Myöhänen E Säätelä T. Parent-child relationship of physical activity patterns and obesity. Int J Obes (1999) 23(12):1262–8. doi: 10.1038/sj.ijo.0801061 10643682

